# The Colony for Epileptics

**Published:** 1897-05-15

**Authors:** 


Vol. XXII.—No. 555.] [May 15, 1897.

### The Colony for Epileptics.

The last public act of Mr. Bayard in England, when he was already divested of the ambassadorial character which he has done so much to adorn, and when he was on the very eve of departure to the great country of which he is so distinguished a citizen, was to lay the foundation-stone of an important addition to one of the most valuable of our medical charities, an institution still in its infancy, but already doing excellent work, and holding forth abundant promise of a long career of public usefulness. The institution to which we refer is the colony or working home for epileptics, at Chalfont St. Peters, in Buckinghamshire; and the addition thus auspiciously commenced is a new home for men, to be erected at the cost of Mr. Passmore Edwards, who has already given the site for the colony, and the home for women which Mrs. Bayard declared open on the same occasion. The colony has come into existence very much as a result of representations made by the physicians to the National Hospital for the Paralysed and Epileptic, in Queen Square, Bloomsbury; and its claims for support were first brought before the public in a meeting held at the Mansion House, at which the late Sir Andrew Clark was one of the most emphatic and most convincing speakers. All that he advanced with regard to the merits of a project which was then only in contemplation has been amply justified by the record of its operations, even during the short time which it has as yet been in existence.

The value of the "colony" rests upon two grounds, distinct although related, one of which is of a purely medical character, while the other might be described as compassionate. The medical fact on which the former depends is that strenuous muscular work tends powerfully to keep epilepsy at bay. Whatever the explanation of this may be, there can be no doubt as to the reality of the influence. The difficulty of turning it to practical account is often considerable, and depends partly upon the danger of leaving an epileptic to work alone, and partly upon the not unnatural unwillingness of employers to afford him any opportunity of working in com bination with others. It is only in comparatively rare cases that an impending fit gives any useful or recognisable warning, and, as a rule, even its earliest stage is attended by too much impairment of consciousness to permit the unfortunate subject to take any precautions for the purpose of saving himself from injury. He is liable to fall from a height, or into a fire, or into water, or to wound himself with the tools with which he was working; and on all these grounds it is not safe to permit him to be alone. If he be among others, the occurrence of a fit disturbs and alarms them all; and produces a general suspension of activity such as an employer, however kindly or indulgent, can hardly be expected to tolerate as a matter of frequent or even occasional recurrence. The general consequence is, not only that the epileptic cannot find employment at his trade, whatever it may be, but also that he cannot safely be left alone, and hence that some other member of the household to which he belongs is withdrawn from remunerative occupation in order to be his companion and guard. The epileptic is thus a double burden upon his or her family; and, thoroughly accustomed as medical men become to the reciprocal kindliness and helpfulness of the really or comparatively poor, yet the examples of these which are seen in relation to epilepsy are so noteworthy as to call for frequent remark. The unfortunate sufferer is attended in his walks to and from the hospital, or in the daily exercise essential to his health, with constant and uncomplaining assiduity; insomuch that, notwithstanding the lamentable frequency of the disease, deaths, or serious injuries arising from accidents during a fit are certainly of somewhat rare occurrence.

The colony at Chalfont St. Peters aims at providing for the inmates, who may be either poor, and maintained there by charity and by the results of their labour, or comparatively rich and maintained out of their own resources, such daily opportunities of active muscular exertion, or, in other words, of becoming thoroughly tired, as experience has shown to be highly beneficial in warding off their attacks, or even in doing away with their liability to them; while, at the same time, this advantage is enjoyed in a community of mutually watchful persons, each of whom is too well acquainted with the character of the fits to be causelessly alarmed by them, and each of whom is ready to render, to anyone who may be attacked, the trifling services which he who renders them knows he may at any time require from others for himself. During the short time that the institution has existed, the results of the industrial life, regarded as a means of medical treatment, have been in the highest degree satisfactory, and it seems probable not only that epileptics of the wealthier classes will soon be seeking to participate in the benefits of the colony, but also that experience will point to the necessity of establishing similar institutions in other counties. They would change the present hapless condition of epileptics into one of comparative happiness and security, attended by a greatly increased prospect of recovery.

